# Crystal structure of *trans*-di­chloridobis­[*N*-(5,5-di­methyl-4,5-di­hydro-3*H*-pyrrol-2-yl-κ*N*)acetamide]palladium(II) dihydrate

**DOI:** 10.1107/S2056989017003929

**Published:** 2017-03-17

**Authors:** Jamal Lasri, Naser Eltaher Eltayeb, Matti Haukka, Bandar A. Babgi

**Affiliations:** a Department of Chemistry, Rabigh College of Science and Arts, PO Box 344, King Abdulaziz University, Jeddah, Saudi Arabia; b University of Jyväskylä, Department of Chemistry, University of Jyväskylä, PO Box 35, FI-40014, Finland

**Keywords:** crystal structure, palladium, nitrile, nitrone, cyclo­addition, N—O bond cleavage

## Abstract

The title complex, [PdCl_2_(C_8_H_14_N_2_O)_2_]·2H_2_O, was obtained by N–O bond cleavage of the oxa­diazo­line rings of the *trans*-[di­chlorido-bis­(2,5,5-trimethyl-5,6,7,7a-tetra­hydro­pyrrolo­[1,2-*b*][1,2,4]oxa­diazole-*N* ^1^)]palladium(II) complex. The palladium(II) atom exhibits an almost square-planar coordination provided by two *trans*-arranged chloride anions and a nitro­gen atom from each of the two neutral organic ligands. In the crystal, N—H⋯O, O—H⋯O and O—H⋯Cl hydrogen bonds link complex mol­ecules into double layers parallel to the *bc* plane.

## Abstract 2

The synthesis and crystal structure of the complex *trans*-[di­chlorido-bis­(*N*-(4,5-di­hydro-5,5-dimethyl-3*H*-pyrrol-2-yl)acetamide)]palladium(II) dihydrate is described.

## Chemical context   

The [2 + 3]-cyclo­addition of nitro­nes with nitriles is one of the most important routes for the synthesis of 1,2,4-oxa­diazo­lines (Bokach *et al.*, 2011 ▸). However, there are some limitations for this method, as only electrophilically activated nitriles react with nitro­nes under harsh conditions and/or long reaction times (Eberson *et al.*, 1998 ▸; Lasri *et al.*, 2008 ▸). The coordination of nitriles to a suitable metal atom becomes a convenient methodology and facile metal-mediated route for the synthesis of a large number of compounds, inaccessible directly by pure organic chemistry (Bokach *et al.*, 2011 ▸). The N—O bond cleavage of oxa­diazo­line rings can be promoted by thermal heating to furnish the derived keto­imine complexes (Lasri *et al.*, 2011 ▸). Moreover, the oxa­diazo­line ligands are opened by N—O bond cleavage to form pyrrolylbenzamide derivatives in which the N atoms of the pyrrolyl moieties coordinate to the palladium atom in the *trans* positions (Lasri *et al.*, 2009 ▸).

In this work, we report the synthesis and crystal structure of the title complex *trans*-[di­chlorido-bis­(*N*-(4,5-di­hydro-5,5-dimethyl-3*H*-pyrrol-2-yl)acetamide)]palladium(II) dihydrate, **2**.

The fused bicyclic 1,2,4-oxa­diazo­line palladium(II) complex *trans-*[PdCl_2_{N=C(Me)ONC(H)CH_2_CH_2_CMe_2_}_2_] (**1**) was previously synthesized by one of us (Lasri *et al.*, 2009 ▸), in good yield (*ca* 75%), by treatment of *trans*-[PdCl_2_(NCMe)_2_] with pyrroline *N*-oxide ^−^O^+^N=CHCH_2_CH_2_CMe_2_ (Scheme, reaction *a*). Inter­estingly, refluxing complex **1** in CHCl_3_ for one week affords a mixture of compounds from which the title compound **2** was isolated by mechanical separation of the crystals obtained from slow evaporation of an acetone/toluene (30:1 *v*/*v*) solution. Compound **2** was characterized by IR spectroscopy and also by X-ray diffraction, which shows that the oxa­diazo­line ligands of **1** have opened by N—O bond cleavage to form a pyrrolylacetamide derivative, *i.e. N*-(4,5-di­hydro-5,5-dimethyl-3*H*-pyrrol-2-yl)acetamide, in which the *N*-atoms of the pyrrolyl moieties coordinate to palladium in the *trans* position (Scheme, reaction *b*).
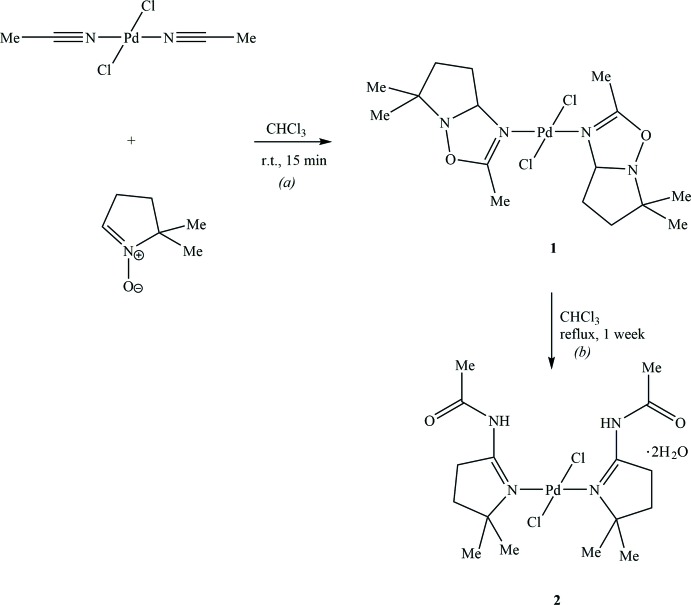


## Structural commentary   

**Figure 1:**
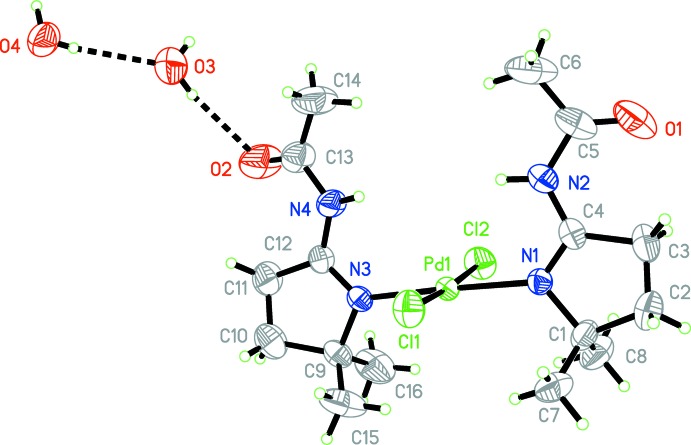
The mol­ecular structure of the title compound with displacement ellipsoids drawn at the 50% probability level. Dashed lines indicate hydrogen bonds

The slightly distorted square-planar coordination sphere around the Pd^II^ atom comprises two chloride anions and two nitro­gen atoms from two neutral organic ligands (Fig. 1 ▸). The Cl—Pd—Cl, N—Pd—N, and Cl—Pd—N angles all deviate by less that 5° from the ideal 90° or 180° angles. The Pd—N [mean value 2.0783 (16) Å] and Pd—Cl [mean value 2.336 (12) Å] bond lengths fall in the range of typical distances found in similar types of Pd^II^ complexes. The five-membered heterocyclic rings each have a twist conformation, with puckering parameters *Q* = 0.238 (4) Å, *φ* = −108.8 (8)° and Q = 0.245 (4) Å, *φ* = 69.9 (8)° for N1/C1–C4 and N3/C9–C12, respectively. The crystal structures of the 2-ethyl and 2-(4-bromo­phen­yl) analogues of the title compounds have been reported elsewhere (Lasri *et al.*, 2009 ▸).

## Supra­molecular features   

**Figure 2:**
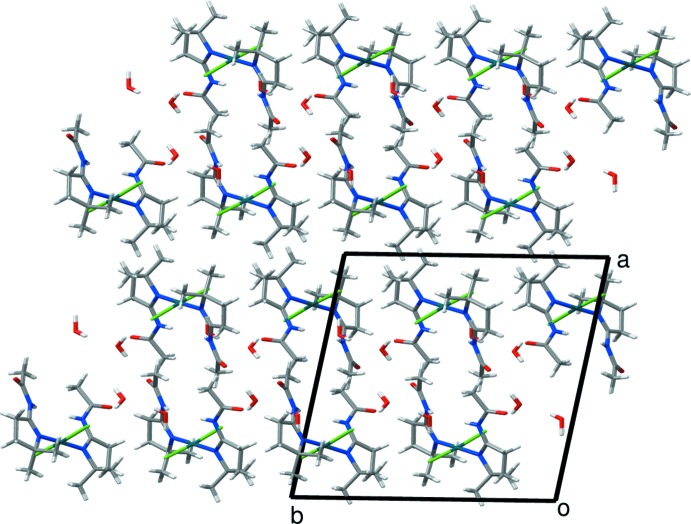
Packing diagram of the title compound viewed down the *c* axis.

In the asymmetric unit, both the N2 and N4 atoms act as hydrogen-bond donors for the O3 atom of a water mol­ecule (Table 1 ▸). The water mol­ecule including the O3 atom also acts as a hydrogen-bond donor to Cl2 and to a second water mol­ecule (O4) which, in turn, forms hydrogen bonds with the Cl1 and O3 atoms of neighboring metal complexes. A view of the crystal packing (Fig. 2 ▸) shows that the mol­ecules are organized in such a way that hydrogen bonds form double layers of metal complexes parallel to the *bc* plane, mainly connected by weak van der Waals inter­actions.

## Synthesis and crystallization   

A solution of bis­(1,2,4-oxa­diazo­line) palladium(II) (complex **1**; 100 mg, 0.206 mmol; Lasri *et al.*, 2009 ▸) in CHCl_3_ (10 mL) was refluxed for one week. The solvent was then removed *in vacuo* and the resulting solid was washed with three 10 mL portions of diethyl ether and dried under air to give a yellow solid. The ^1^H and ^13^C NMR spectra in CDCl_3_ of the obtained solid show the presence of a mixture of compounds. However, the pyrrolylacetamide product **2** was isolated by mechanical separation of the crystals obtained from slow evaporation of an acetone/toluene (30:1 *v*/*v*) solution. The IR spectrum of **2** shows strong ν(NC=O) and ν(N=C) vibrations at 1729 and 1644 cm^−1^, respectively, and ν(NH) at 3300 cm^−1^.

## Refinement   

Crystal data, data collection and structure refinement details are summarized in Table 2 ▸. The amine and water hydrogen atoms were located in a difference-Fourier map and refined isotropically. All other hydrogen atoms were positioned geometrically and constrained to ride on their parent atoms, with C—H = 0.96–0.97 Å, and with *U*_iso_ = 1.2 *U*_eq_(C) or 1.5*U*_eq_(C) for methyl H atoms. A rotating model was applied to the methyl groups. The maximum electron density is located 0.97 Å from atom Pd1 and the minimum electron density is located 0.95 Å from atom Pd1. Two outliers (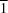
02 and 002) were omitted in the last cycles of refinement.

## Supplementary Material

Crystal structure: contains datablock(s) I. DOI: 10.1107/S2056989017003929/rz5207sup1.cif

Structure factors: contains datablock(s) I. DOI: 10.1107/S2056989017003929/rz5207Isup2.hkl

CCDC reference: 1537327

Additional supporting information:  crystallographic information; 3D view; checkCIF report

## Figures and Tables

**Table 1:** Hydrogen-bond geometry (Å, °)

*D*—H⋯*A*	*D*—H	H⋯*A*	*D*⋯*A*	*D*—H⋯*A*
O4—H1⋯Cl1^i^	0.98 (10)	1.99 (9)	2.952 (4)	169 (8)
O4—H2⋯O3	0.69 (3)	2.22 (3)	2.909 (5)	171 (3)
O3—H3⋯Cl2^ii^	0.71 (6)	2.58 (7)	3.269 (5)	163 (8)
O3—H4⋯O2	0.81 (7)	2.19 (7)	2.994 (6)	169 (7)
N4—H5⋯O4^iii^	0.87 (3)	2.19 (4)	3.010 (5)	159 (3)
N2—H6⋯O4^iii^	0.88 (3)	2.40 (3)	3.194 (5)	151 (3)

Symmetry codes: (i) 
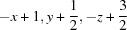
; (ii) 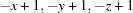; (iii) 
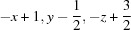
.

**Table 2:** Experimental details

Crystal data	
Chemical formula	[PdCl_2_(C_8_H_14_N_2_O)_2_]·2H_2_O
*M*_r_	521.75
Crystal system, space group	Monoclinic, *P*2_1_/*c*
Temperature (K)	293
*a*, *b*, *c* (Å)	15.945 (12), 8.765 (6), 16.894 (13)
β (°)	101.481 (19)
*V* (Å^3^)	2314 (3)
*Z*	4
Radiation type	Mo *K*α
μ (mm^−1^)	1.06
Crystal size (mm)	0.24 × 0.20 × 0.05

Data collection	
Diffractometer	Bruker D8 Quest
Absorption correction	Multi-scan (*SADABS*; Bruker, 2016 ▸); additional spherical absorption correction applied with μ*r = 0.2000
*T*_min_, *T*_max_	0.594, 0.745
No. of measured, independent and observed [*I* > 2σ(*I*)] reflections	30712, 4239, 3546
*R*_int_	0.050
(sin θ/λ)_max_ (Å^−1^)	0.637

Refinement	
*R*[*F* ^2^ > 2σ(*F* ^2^)], *wR*(*F* ^2^), *S*	0.032, 0.071, 1.07
No. of reflections	4239
No. of parameters	274
H-atom treatment	H atoms treated by a mixture of independent and constrained refinement
Δρ_max_, Δρ_min_ (e Å^−3^)	0.56, −0.50

Computer programs: *APEX3* and *SAINT* (Bruker, 2016 ▸), *SHELXS2014/7* (Sheldrick, 2008 ▸), *SHELXL2014/7* (Sheldrick, 2015 ▸) and *PLATON* (Spek, 2015 ▸).

## supplementary crystallographic information

### Crystal data

**Table:**

[PdCl_2_(C_8_H_14_N_2_O)_2_]·2H_2_O	*F*(000) = 1072
*M**_r_* = 521.75	*D*_x_ = 1.498 Mg m^−^^3^
Monoclinic, *P*2_1_/*c*	Mo *K*α radiation, λ = 0.71073 Å
*a* = 15.945 (12) Å	Cell parameters from 9661 reflections
*b* = 8.765 (6) Å	θ = 2.5–25.4°
*c* = 16.894 (13) Å	µ = 1.06 mm^−^^1^
β = 101.481 (19)°	*T* = 293 K
*V* = 2314 (3) Å^3^	Block, yellow
*Z* = 4	0.24 × 0.20 × 0.05 mm

### Data collection

**Table:**

Bruker D8 Quest diffractometer	3546 reflections with *I* > 2σ(*I*)
Radiation source: sealed X-Ray tube	*R*_int_ = 0.050
φ and ω scans	θ_max_ = 26.9°, θ_min_ = 2.6°
Absorption correction: multi-scan (SADABS; Bruker, 2016); additional spherical absorption correction applied with µ**r* = 0.2000	*h* = −20→20
*T*_min_ = 0.594, *T*_max_ = 0.745	*k* = −10→10
30712 measured reflections	*l* = −18→19
4239 independent reflections	

### Refinement

**Table:**

Refinement on *F*^2^	0 restraints
Least-squares matrix: full	Hydrogen site location: mixed
*R*[*F*^2^ > 2σ(*F*^2^)] = 0.032	H atoms treated by a mixture of independent and constrained refinement
*wR*(*F*^2^) = 0.071	*w* = 1/[σ^2^(*F*_o_^2^) + (0.0253*P*)^2^ + 2.688*P*] where *P* = (*F*_o_^2^ + 2*F*_c_^2^)/3
*S* = 1.07	(Δ/σ)_max_ = 0.001
4239 reflections	Δρ_max_ = 0.56 e Å^−^^3^
274 parameters	Δρ_min_ = −0.50 e Å^−^^3^

### Special details

**Table:**

Geometry. All esds (except the esd in the dihedral angle between two l.s. planes) are estimated using the full covariance matrix. The cell esds are taken into account individually in the estimation of esds in distances, angles and torsion angles; correlations between esds in cell parameters are only used when they are defined by crystal symmetry. An approximate (isotropic) treatment of cell esds is used for estimating esds involving l.s. planes.

### Fractional atomic coordinates and isotropic or equivalent isotropic displacement parameters (Å^2^)

**Table:**

	*x*	*y*	*z*	*U*_iso_*/*U*_eq_	
Pd1	0.21183 (2)	0.43221 (3)	0.41558 (2)	0.02882 (8)	
Cl1	0.15431 (6)	0.28686 (10)	0.50868 (5)	0.0475 (2)	
Cl2	0.27918 (6)	0.56600 (11)	0.32714 (6)	0.0523 (2)	
O1	0.3558 (2)	−0.0129 (5)	0.2615 (2)	0.1043 (13)	
O2	0.46851 (17)	0.6655 (3)	0.6391 (2)	0.0817 (10)	
O3	0.6085 (3)	0.6187 (6)	0.7839 (2)	0.0918 (13)	
O4	0.6653 (2)	0.6870 (4)	0.9547 (2)	0.0641 (8)	
N1	0.18404 (15)	0.2675 (3)	0.32579 (15)	0.0330 (6)	
N2	0.30573 (19)	0.1373 (3)	0.3545 (2)	0.0458 (7)	
N3	0.23602 (16)	0.6039 (3)	0.50208 (15)	0.0330 (6)	
N4	0.36553 (17)	0.5252 (4)	0.56263 (18)	0.0438 (7)	
C1	0.1043 (2)	0.2662 (4)	0.2637 (2)	0.0432 (8)	
C2	0.1062 (3)	0.1119 (5)	0.2189 (2)	0.0575 (10)	
H2A	0.0740	0.0350	0.2415	0.069*	
H2B	0.0816	0.1233	0.1619	0.069*	
C3	0.1936 (3)	0.0699 (4)	0.2301 (2)	0.0528 (9)	
H3A	0.2186	0.1003	0.1847	0.063*	
H3B	0.2016	−0.0388	0.2393	0.063*	
C4	0.2301 (2)	0.1634 (4)	0.30675 (19)	0.0368 (7)	
C5	0.3638 (3)	0.0430 (5)	0.3314 (3)	0.0642 (11)	
C6	0.4363 (3)	0.0199 (6)	0.4007 (3)	0.0914 (17)	
H6A	0.4398	0.1053	0.4368	0.137*	
H6B	0.4887	0.0114	0.3812	0.137*	
H6C	0.4271	−0.0718	0.4288	0.137*	
C7	0.0348 (2)	0.2747 (5)	0.3112 (3)	0.0652 (12)	
H7A	−0.0198	0.2642	0.2754	0.098*	
H7B	0.0374	0.3714	0.3383	0.098*	
H7C	0.0420	0.1940	0.3504	0.098*	
C8	0.1026 (3)	0.3988 (5)	0.2027 (2)	0.0623 (11)	
H8A	0.0500	0.3957	0.1636	0.093*	
H8B	0.1499	0.3884	0.1757	0.093*	
H8C	0.1070	0.4944	0.2309	0.093*	
C9	0.1786 (2)	0.7343 (4)	0.5045 (2)	0.0430 (8)	
C10	0.2181 (3)	0.8178 (5)	0.5865 (3)	0.0696 (12)	
H10A	0.2118	0.9275	0.5808	0.084*	
H10B	0.1904	0.7841	0.6297	0.084*	
C11	0.3071 (2)	0.7748 (4)	0.6028 (2)	0.0557 (10)	
H11A	0.3284	0.7564	0.6599	0.067*	
H11B	0.3423	0.8515	0.5839	0.067*	
C12	0.3034 (2)	0.6287 (4)	0.55392 (19)	0.0363 (7)	
C13	0.4437 (2)	0.5445 (5)	0.6085 (2)	0.0547 (10)	
C14	0.4948 (2)	0.4047 (5)	0.6150 (3)	0.0798 (15)	
H14A	0.4739	0.3403	0.5693	0.120*	
H14B	0.4905	0.3519	0.6638	0.120*	
H14C	0.5535	0.4303	0.6160	0.120*	
C15	0.0952 (3)	0.6747 (5)	0.5097 (3)	0.0810 (14)	
H15A	0.0571	0.7578	0.5139	0.121*	
H15B	0.1002	0.6107	0.5565	0.121*	
H15C	0.0729	0.6162	0.4621	0.121*	
C16	0.1746 (3)	0.8313 (5)	0.4258 (3)	0.0782 (14)	
H16A	0.1352	0.9143	0.4253	0.117*	
H16B	0.1556	0.7680	0.3793	0.117*	
H16C	0.2304	0.8709	0.4245	0.117*	
H1	0.727 (6)	0.706 (10)	0.968 (5)	0.23 (4)*	
H2	0.647 (2)	0.669 (4)	0.915 (2)	0.036 (12)*	
H3	0.641 (4)	0.588 (8)	0.765 (4)	0.12 (3)*	
H4	0.570 (5)	0.644 (8)	0.747 (4)	0.15 (3)*	
H5	0.353 (2)	0.434 (4)	0.544 (2)	0.036 (9)*	
H6	0.316 (2)	0.186 (4)	0.401 (2)	0.044 (10)*	

### Atomic displacement parameters (Å^2^)

**Table:**

	*U*^11^	*U*^22^	*U*^33^	*U*^12^	*U*^13^	*U*^23^
Pd1	0.02776 (12)	0.02964 (13)	0.02942 (14)	0.00102 (10)	0.00656 (9)	−0.00173 (11)
Cl1	0.0576 (5)	0.0483 (5)	0.0396 (5)	−0.0088 (4)	0.0168 (4)	0.0040 (4)
Cl2	0.0484 (5)	0.0583 (5)	0.0565 (5)	−0.0123 (4)	0.0260 (4)	−0.0003 (5)
O1	0.101 (3)	0.116 (3)	0.101 (3)	0.047 (2)	0.034 (2)	−0.027 (2)
O2	0.0476 (16)	0.064 (2)	0.125 (3)	−0.0168 (15)	−0.0023 (17)	−0.0192 (19)
O3	0.076 (3)	0.122 (3)	0.072 (2)	0.015 (2)	0.002 (2)	−0.035 (2)
O4	0.063 (2)	0.065 (2)	0.058 (2)	0.0002 (15)	−0.0035 (16)	−0.0105 (17)
N1	0.0315 (13)	0.0333 (14)	0.0344 (14)	−0.0017 (11)	0.0070 (11)	−0.0043 (12)
N2	0.0457 (17)	0.0401 (17)	0.0516 (19)	0.0085 (13)	0.0100 (15)	−0.0064 (15)
N3	0.0363 (14)	0.0281 (14)	0.0359 (14)	0.0023 (11)	0.0102 (12)	−0.0004 (11)
N4	0.0343 (15)	0.0404 (18)	0.0538 (19)	−0.0045 (13)	0.0018 (13)	−0.0087 (14)
C1	0.0386 (18)	0.050 (2)	0.0368 (18)	−0.0045 (15)	−0.0020 (14)	−0.0042 (16)
C2	0.062 (2)	0.057 (2)	0.050 (2)	−0.0137 (19)	0.0011 (18)	−0.0125 (19)
C3	0.074 (3)	0.044 (2)	0.041 (2)	0.0026 (19)	0.0120 (18)	−0.0076 (17)
C4	0.0472 (19)	0.0310 (17)	0.0346 (18)	−0.0012 (15)	0.0139 (15)	0.0006 (14)
C5	0.061 (3)	0.048 (2)	0.089 (3)	0.016 (2)	0.030 (2)	0.002 (2)
C6	0.053 (3)	0.079 (3)	0.140 (5)	0.027 (2)	0.014 (3)	0.007 (3)
C7	0.0337 (19)	0.087 (3)	0.071 (3)	−0.003 (2)	0.0012 (18)	−0.006 (2)
C8	0.060 (2)	0.059 (3)	0.058 (2)	0.001 (2)	−0.0113 (19)	0.006 (2)
C9	0.048 (2)	0.0326 (18)	0.050 (2)	0.0088 (15)	0.0162 (16)	−0.0012 (15)
C10	0.081 (3)	0.050 (2)	0.077 (3)	0.013 (2)	0.015 (2)	−0.021 (2)
C11	0.064 (3)	0.039 (2)	0.061 (2)	−0.0030 (18)	0.0060 (19)	−0.0127 (18)
C12	0.0408 (18)	0.0303 (16)	0.0384 (18)	−0.0032 (14)	0.0088 (15)	−0.0016 (14)
C13	0.0393 (19)	0.057 (3)	0.066 (3)	−0.0099 (18)	0.0078 (18)	−0.003 (2)
C14	0.036 (2)	0.074 (3)	0.122 (4)	0.003 (2)	0.000 (2)	−0.010 (3)
C15	0.053 (3)	0.066 (3)	0.130 (4)	0.020 (2)	0.034 (3)	−0.001 (3)
C16	0.099 (3)	0.061 (3)	0.080 (3)	0.039 (3)	0.029 (3)	0.025 (2)

### Geometric parameters (Å, º)

**Table:**

Pd1—N1	2.077 (3)	C5—C6	1.486 (6)
Pd1—N3	2.080 (3)	C6—H6A	0.9600
Pd1—Cl2	2.3238 (14)	C6—H6B	0.9600
Pd1—Cl1	2.3478 (14)	C6—H6C	0.9600
O1—C5	1.262 (5)	C7—H7A	0.9600
O2—C13	1.211 (5)	C7—H7B	0.9600
O3—H3	0.71 (6)	C7—H7C	0.9600
O3—H4	0.81 (7)	C8—H8A	0.9600
O4—H1	0.98 (9)	C8—H8B	0.9600
O4—H2	0.70 (4)	C8—H8C	0.9600
N1—C4	1.253 (4)	C9—C15	1.448 (5)
N1—C1	1.478 (4)	C9—C16	1.569 (5)
N2—C4	1.330 (4)	C9—C10	1.583 (5)
N2—C5	1.355 (5)	C10—C11	1.442 (5)
N2—H6	0.88 (3)	C10—H10A	0.9700
N3—C12	1.263 (4)	C10—H10B	0.9700
N3—C9	1.470 (4)	C11—C12	1.518 (5)
N4—C12	1.329 (4)	C11—H11A	0.9700
N4—C13	1.341 (5)	C11—H11B	0.9700
N4—H5	0.87 (3)	C13—C14	1.463 (6)
C1—C7	1.494 (5)	C14—H14A	0.9600
C1—C8	1.551 (5)	C14—H14B	0.9600
C1—C2	1.553 (5)	C14—H14C	0.9600
C2—C3	1.417 (5)	C15—H15A	0.9600
C2—H2A	0.9700	C15—H15B	0.9600
C2—H2B	0.9700	C15—H15C	0.9600
C3—C4	1.545 (5)	C16—H16A	0.9600
C3—H3A	0.9700	C16—H16B	0.9600
C3—H3B	0.9700	C16—H16C	0.9600
			
N1—Pd1—N3	177.41 (10)	H7A—C7—H7B	109.5
N1—Pd1—Cl2	86.56 (9)	C1—C7—H7C	109.5
N3—Pd1—Cl2	92.39 (9)	H7A—C7—H7C	109.5
N1—Pd1—Cl1	93.26 (9)	H7B—C7—H7C	109.5
N3—Pd1—Cl1	87.97 (9)	C1—C8—H8A	109.5
Cl2—Pd1—Cl1	175.38 (3)	C1—C8—H8B	109.5
H3—O3—H4	105 (7)	H8A—C8—H8B	109.5
H1—O4—H2	117 (6)	C1—C8—H8C	109.5
C4—N1—C1	106.2 (3)	H8A—C8—H8C	109.5
C4—N1—Pd1	130.0 (2)	H8B—C8—H8C	109.5
C1—N1—Pd1	123.5 (2)	C15—C9—N3	107.8 (3)
C4—N2—C5	121.3 (4)	C15—C9—C16	111.3 (4)
C4—N2—H6	117 (2)	N3—C9—C16	108.5 (3)
C5—N2—H6	122 (2)	C15—C9—C10	108.9 (3)
C12—N3—C9	107.2 (3)	N3—C9—C10	104.0 (3)
C12—N3—Pd1	128.7 (2)	C16—C9—C10	115.7 (3)
C9—N3—Pd1	123.6 (2)	C11—C10—C9	104.5 (3)
C12—N4—C13	124.3 (3)	C11—C10—H10A	110.8
C12—N4—H5	119 (2)	C9—C10—H10A	110.8
C13—N4—H5	116 (2)	C11—C10—H10B	110.8
N1—C1—C7	104.1 (3)	C9—C10—H10B	110.8
N1—C1—C8	111.4 (3)	H10A—C10—H10B	108.9
C7—C1—C8	113.8 (3)	C10—C11—C12	100.8 (3)
N1—C1—C2	104.7 (3)	C10—C11—H11A	111.6
C7—C1—C2	113.1 (3)	C12—C11—H11A	111.6
C8—C1—C2	109.2 (3)	C10—C11—H11B	111.6
C3—C2—C1	106.0 (3)	C12—C11—H11B	111.6
C3—C2—H2A	110.5	H11A—C11—H11B	109.4
C1—C2—H2A	110.5	N3—C12—N4	118.2 (3)
C3—C2—H2B	110.5	N3—C12—C11	117.2 (3)
C1—C2—H2B	110.5	N4—C12—C11	124.6 (3)
H2A—C2—H2B	108.7	O2—C13—N4	123.0 (4)
C2—C3—C4	99.8 (3)	O2—C13—C14	124.9 (4)
C2—C3—H3A	111.8	N4—C13—C14	112.1 (3)
C4—C3—H3A	111.8	C13—C14—H14A	109.5
C2—C3—H3B	111.8	C13—C14—H14B	109.5
C4—C3—H3B	111.8	H14A—C14—H14B	109.5
H3A—C3—H3B	109.5	C13—C14—H14C	109.5
N1—C4—N2	118.5 (3)	H14A—C14—H14C	109.5
N1—C4—C3	117.3 (3)	H14B—C14—H14C	109.5
N2—C4—C3	124.2 (3)	C9—C15—H15A	109.5
O1—C5—N2	124.0 (4)	C9—C15—H15B	109.5
O1—C5—C6	127.1 (4)	H15A—C15—H15B	109.5
N2—C5—C6	109.0 (4)	C9—C15—H15C	109.5
C5—C6—H6A	109.5	H15A—C15—H15C	109.5
C5—C6—H6B	109.5	H15B—C15—H15C	109.5
H6A—C6—H6B	109.5	C9—C16—H16A	109.5
C5—C6—H6C	109.5	C9—C16—H16B	109.5
H6A—C6—H6C	109.5	H16A—C16—H16B	109.5
H6B—C6—H6C	109.5	C9—C16—H16C	109.5
C1—C7—H7A	109.5	H16A—C16—H16C	109.5
C1—C7—H7B	109.5	H16B—C16—H16C	109.5
			
C4—N1—C1—C7	133.6 (3)	C12—N3—C9—C15	−130.9 (3)
Pd1—N1—C1—C7	−52.9 (4)	Pd1—N3—C9—C15	57.0 (4)
C4—N1—C1—C8	−103.4 (3)	C12—N3—C9—C16	108.4 (4)
Pd1—N1—C1—C8	70.2 (3)	Pd1—N3—C9—C16	−63.7 (4)
C4—N1—C1—C2	14.5 (3)	C12—N3—C9—C10	−15.3 (4)
Pd1—N1—C1—C2	−171.9 (2)	Pd1—N3—C9—C10	172.5 (2)
N1—C1—C2—C3	−24.4 (4)	C15—C9—C10—C11	139.3 (4)
C7—C1—C2—C3	−137.2 (3)	N3—C9—C10—C11	24.6 (4)
C8—C1—C2—C3	95.0 (4)	C16—C9—C10—C11	−94.4 (4)
C1—C2—C3—C4	22.3 (4)	C9—C10—C11—C12	−22.5 (4)
C1—N1—C4—N2	−178.3 (3)	C9—N3—C12—N4	−179.9 (3)
Pd1—N1—C4—N2	8.7 (5)	Pd1—N3—C12—N4	−8.3 (5)
C1—N1—C4—C3	−0.2 (4)	C9—N3—C12—C11	0.9 (4)
Pd1—N1—C4—C3	−173.2 (2)	Pd1—N3—C12—C11	172.4 (2)
C5—N2—C4—N1	−169.9 (3)	C13—N4—C12—N3	172.2 (3)
C5—N2—C4—C3	12.1 (5)	C13—N4—C12—C11	−8.6 (6)
C2—C3—C4—N1	−15.3 (4)	C10—C11—C12—N3	15.3 (4)
C2—C3—C4—N2	162.7 (3)	C10—C11—C12—N4	−163.9 (4)
C4—N2—C5—O1	8.2 (7)	C12—N4—C13—O2	−8.9 (6)
C4—N2—C5—C6	−172.4 (4)	C12—N4—C13—C14	172.6 (4)

### Hydrogen-bond geometry (Å, º)

**Table:**

*D*—H···*A*	*D*—H	H···*A*	*D*···*A*	*D*—H···*A*
O4—H1···Cl1^i^	0.98 (10)	1.99 (9)	2.952 (4)	169 (8)
O4—H2···O3	0.69 (3)	2.22 (3)	2.909 (5)	171 (3)
O3—H3···Cl2^ii^	0.71 (6)	2.58 (7)	3.269 (5)	163 (8)
O3—H4···O2	0.81 (7)	2.19 (7)	2.994 (6)	169 (7)
N4—H5···O4^iii^	0.87 (3)	2.19 (4)	3.010 (5)	159 (3)
N2—H6···O4^iii^	0.88 (3)	2.40 (3)	3.194 (5)	151 (3)

Symmetry codes: (i) −*x*+1, *y*+1/2, −*z*+3/2; (ii) −*x*+1, −*y*+1, −*z*+1; (iii) −*x*+1, *y*−1/2, −*z*+3/2.
